# Work-Related Disorders in Public Transportation Drivers and the Length of Exposure

**DOI:** 10.3390/jcm14145018

**Published:** 2025-07-15

**Authors:** Florina Georgeta Popescu, Corina Bolocan, Manuela Oancea, Iulia Iovanca Drăgoi, Nicolae Herisanu, Corina Oancea, Nilima Rajpal Kundnani, Claudia Mariana Handra, Marina Ruxandra Oțelea, Dan Alexandru Surducan

**Affiliations:** 1 Department 5, “Victor Babes” University of Medicine and Pharmacy, 2 Eftimie Murgu Square, 300041 Timisoara, Romania; popescu.florina@umft.ro; 2 SCM-Profilaxis SRL, 106, 1 Decembrie 1918 Street, 300566 Timisoara, Romania; corinabolocan@gmail.com; 3 Centrul Medical Explomed, 39A Lugojului Street, 300305 Timisoara, Romania; oancea_manu@yahoo.com; 4 Fast Fizio Clinic, 45 Olanda Street, 300261 Timisoara, Romania; contactfastfizioclinic@gmail.com; 5 Faculty of Mechanics, Politehnica University Timisoara, 2 Mihai Viteazu Bd, 300222 Timisoara, Romania; n_herisanu@yahoo.co.uk; 6 Clinical Department 9, University of Medicine and Pharmacy Carol Davila, 37, Dionisie Lupu St., 030167 Bucharest, Romania; corina.oancea@umfcd.ro; 7 Department 6, Cardiology, University Clinic of Internal Medicine and Ambulatory Care, Prevention and Cardiovascular Recovery, “Victor Babes” University of Medicine and Pharmacy, 2 Eftimie Murgu Square, 300041 Timisoara, Romania; knilima@umft.ro; 8 Research Centre of Timisoara Institute of Cardiovascular Diseases, “Victor Babes” University of Medicine and Pharmacy, 300041 Timisoara, Romania; 9 Clinical Department 5, University of Medicine and Pharmacy Carol Davila, 37, Dionisie Lupu St., 030167 Bucharest, Romania; claudia.handra@umfcd.ro; 10 Department of Public Health and Health Management, “Victor Babes” University of Medicine and Pharmacy, 2 Eftimie Murgu Square, 300041 Timisoara, Romania; surducan.dan@umft.ro

**Keywords:** drivers, high blood pressure, neck pain, dorsal pain, lumbar pain, tenure

## Abstract

**Background/Objectives**: Public transportation drivers are exposed to a variety of occupational hazards. The scope of this study is to describe the most significant changes in symptoms and work-related disorders in the last decade in a sample of professional drivers from a large Romanian city, and, in particular, the cardio-metabolic and musculoskeletal impact. **Methods**: A retrospective study on 186 professional tram, trolley, and bus drivers from a total number of 344 employed by the company was conducted. The initial values (pre-employment) of the BMI, blood pressure, cholesterol, fasting glycemia, and musculoskeletal complaints were compared to the values of the last employment check-up. **Results**: After an average follow-up period of 11 years, BMI increased from 27.69 (SD = 4.68) to 30.06 (SD = 5.2) (*p* < 0.0001), cholesterol from 201.7 (SD = 39.87) to 212.62 (SD = 42.51), (*p* = 0.04). The number of cases of high blood pressure (25 to 56, *p* < 0.0001) and musculoskeletal complaints increased from 3 initial cases to 26 cases of neck pain (*p* = 0.07), from 2 to 49 cases of dorsal pain (*p* = 0.02), and from 18 to 59 cases of lumbar pain (*p* < 0.0001). High blood pressure and low back pain were significantly correlated with tenure, independent of other factors. **Conclusions**: As tenure is important in the development of cardiovascular and musculoskeletal diseases, specific interventions should be developed in the early stages of the drivers’ career.

## 1. Introduction

Public transportation drivers (trams, trolleys, and buses) are exposed to a variety of occupational hazards: air pollution (micro particles, chemical and biological hazards), noise, static work with awkward positions, repetitive movements, vibrations transmitted to the whole body, and stress [1,2].

Driving is a sedentary job. Ideally, this static activity should be compensated with non-occupational physical activity, for which drivers have, in general, limited time, due to the long and irregular working hours. The level of stress in terms of job strain and effort–reward imbalance reported in this occupation is generally high [3], potentially leading to exhaustion [4]. Due to this complex of job-related exposures, public transportation drivers have a high incidence of musculoskeletal diseases [5], hearing loss [6], cardiovascular disease [7,8], sleep disorders [9], stress and depression [10], gastritis [11], reprotoxic effects [12], and even bladder and lung cancer [13]. A systematic reviews have also highlighted an interaction between some of these factors, for example, a significant impact of the cardio-metabolic risk factors, such as dyslipidemia, hyperglycemia, and high blood pressure on the onset of the lumbar disk degeneration [14], for the neck pain [15], or in research looking at the correlation between sleep apnea and hyperglycemia [16], but specific studies dedicated to the interaction of these hazards (e.g., drivers) are missing.

Efforts conducted to reduce urban air pollution in Romania led to some improvements in O_3_ and PM_10_ PM_10_ exposure [17] but the population weighted average PM_2.5_ and NO_2_ still remain higher than the average European levels. Because, in 2019, the level of pollution with CO_2_/passenger transport was three times higher than the average European level [18], many cities have increased the number of electric vehicles to reduce this source of pollution. Changes have targeted also the working conditions of bus drivers, with a reduction in vibration exposure, at least in vehicles used in Timisoara.

From the general environmental data reported, Timisoara roads generate a day–evening–night level of noise (Lden) > 55 dB for 50% of its inhabitants [19], similar to most urban areas in Europe. This European report the trend was, unfortunately, incremental between 2012 and 2017. Some less extensive measurements support this tendency after 2017 [20].

Currently, there is insufficient longitudinal data on the evolution of the most significant complaints of public transportation drivers. This study will update the information on the most significant changes in the symptoms and work-related disorders in the last decade in a sample of professional drivers from a large city in Romania and the relation between the cardio-metabolic risk factors and the musculo-skeletal complains in this working population.

## 2. Materials and Methods

We conducted a retrospective, longitudinal study on 186 professional tram, trolley, and bus drivers from a total number of 344 employed by the public transportation company from Timisoara. Signed written consent was obtained from all participants. Ethical approval from the University of Medicine and Pharmacy “Victor Babes” Timisoara Ethics Committee was released and registered under the approval number No 23, issued in October 2019.

The inclusion criteria for participating in this study was an existent pre-employment check-up and a regular check-up in 2020, further on referred as “follow-up check-up” and performing the same job during the follow up. We used these criteria in order to have the maximum length of follow up for each person included in the analysis. There were no exclusion criteria for drivers, but no managerial or administrative workers were included. Data were extracted from the medical records by the occupational physician in charge of the surveillance of the employees.

Exposure assessment: Organizational charts were discussed with the management in order to extract relevant data on shift rotations. Noise inside the drivers’ cabin was measured with a Bruel & Kjaer sonometer type 2250, class I for the whole duration of the activity (8 h). Values were expressed as the level of daily exposure to noise (Lex8) and the level of peak acoustic pressure (PAP).

Vibration measurements were performed by a certified laboratory of acoustics and vibrations with a human vibration analyzer, B&K 4447 (serial no. 2570925), for the triaxial accelerometer, B&K 4520 (serial no. 51813), and the connection cable, AO-0693-D_025. The methods used for the assessments fulfill the requirement of the SR ISO 2631-1: 2001 [21] standards for mechanical vibrations and shock. The routes were selected to cover the entire network of transportation. The accelerometer was placed on the driver seat to measure the vibrations on three axis, vertical (z), transversal (x), and longitudinal (y). For each vehicle, three measurements were recorded and computed to obtain the resulting value.

The follow-up period was, on average, 11.99 years, with a SD of 7 years. The clinical data on musculoskeletal complaints were recorded with a Romanian version of the Nordic musculoskeletal questionnaire.

High blood pressure was defined either if two consecutive measurements of the blood pressure found abnormal values or if a diagnosis was already established and the worker was treated for hypertension.

The initial (pre-employment) and the follow-up check-up values of total serum cholesterol and glycaemia were extracted from the medical files. As there was no BMI < 20 kg/m^2^ recorded, neither in the pre-employment nor during the check-ups. The following BMI categories were used: 18.5–24.9 kg/m^2^ = normal weight; 25–29.9 kg/m^2^ = overweight; 30–34.9 kg/m^2^ = obesity class I; 35–39.9 kg/m^2^ = obesity class II; >40 kg/m^2^ = obesity class III. Changes in the category of BMI were noted, either as an increase or as a decrease.

Values of cholesterol > 200 mg/dL were classified as hypercholesterolemia. Impaired fasting glucose (IFG) was defined as fasting glucose between 100 and 125 mg/dL [22].

After checking the normality distribution of the data, the statistical analysis was performed with StatPlus:mac, AnalystSoft Inc. Brandon, FL, USA—a statistical analysis program for macOS Version v8. Average, standard deviation (SD) was computed for numerical variables.

We have structured the analysis in two steps. First, we compared the initial data with the data obtained during the follow-up check-up. This comparison was performed with a Chi square test for the categorical variables and a Mann–Whitney U test for the numerical ones.

In the second step, we evaluated the influence of tenure and considered dependent variables of new onset, post-employment symptoms, diagnosis or metabolic risk factors. In the regression model, we used independent variables including tenure, age, sex, body mass index, and the laboratory data.

## 3. Results

1. Exposure variables

All public transportation drivers worked in the same city, with similar environmental hazards (air pollution, traffic). All worked in 2-day shifts (morning and afternoon), with a duration between 8 and 10 h, with some irregularities of working hours (1–2 h hours difference in the starting of working day). After 5 days of work, 2 consecutive resting days followed.

The mean value of the daily vibration exposure was 0.252 m/s^2^, with a (SD = 0.091). We observed higher values of vibrations measurements transmitted to the whole body for the bus drivers, 0.308 m/s^2^, followed by the trolleybus drivers, 0.248 m/s^2^, and then the tramway drivers, 0.164 m/s^2^, but none of them exceeded the legal limit (0.5 m/s^2^). The mean noise level (Lex8) was 70.78 ± 3.93 dB (A) for tram drivers, 69.08 ± 3.12 dB (A) for bus drivers, and 71.49 ± 1.49 dB (A) for trolley drivers (*p* = 0.33). The PAP varied between 107.59 and 115.02 dB (C) in trams, 113.86 and 121.9 dB (C) in buses, and 113.55 and 120.12 dB (C) in trolley buses.

2. General data about the sample

There were 63 bus drivers out of a total of 140 (45%), 62 trolleybus drivers out of 90 (68.89%), and 61 tram drivers out of 114 (42.36%), who had documented medical history, clinical examination, and lab test from the pre-employment exam. From the 186 professional drivers included in the analysis, 25 (13.44%) were women and 161 (85.66%) were men, reflecting the gender structure of the company’s employees. In women, the distribution according to the type of vehicle was the following: 16 tram drivers (64%), 8 trolley drivers (32%), and 1 bus driver (4%). In the group of men, 45 (27.95%) were tram drivers, 54 (33.54%) were trolley drivers, and 62 (38.51%) bus drivers. The difference was statistically significant (chi^2^ = 16.2, *p* = 0.0003).

The average age at the pre-employment check-up was 36.8 (SD = 9.36), and at the follow-up examination it was 48.78 years (SD = 8.38). The average tenure was 11.99 years (SD = 7.01). There were 27 drivers with tenures < 5 years, 32 with tenures between 6 and 10 years, 34 with tenures between 11 and 15 years, and 14 with tenures > 15 years.

3. Comparison of the health characteristics between pre-employment and the follow-up check-up

**Figure 1:**
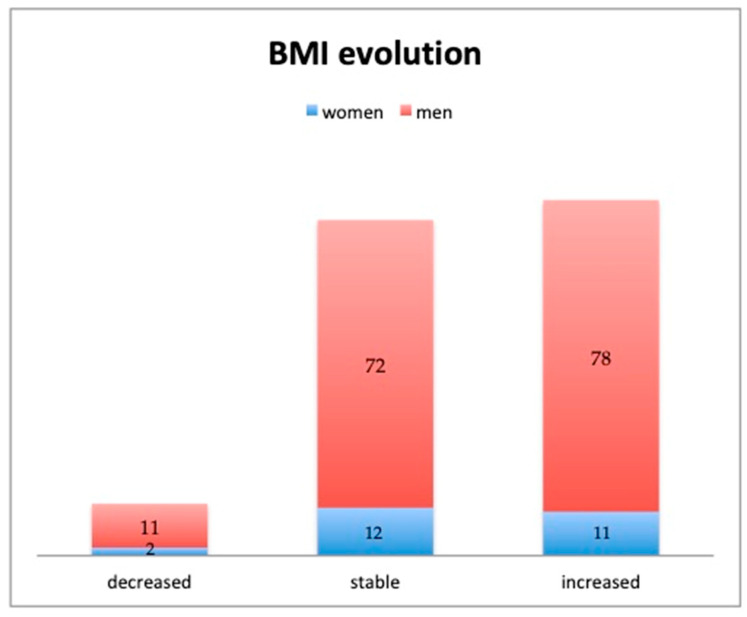
Gender distribution of the number of drivers according to the evolution of the categories of BMI from the pre-employment values.

After an average follow-up period of 11 years, there were several modifications compared to the pre-employment medical consultation (Table 1). For example, compared to pre-employment, 13 drivers (6.98%) decreased and 89 drivers (47.85%) increased their category of BMI, while 84 drivers (45.16%) maintained the same category of BMI (Figure 1). No difference was noted in this variation according to sex (chi^2^ = 0.18, *p* = 0.91) or type of vehicles (chi^2^ = 3.02, *p* = 0.55). The BMI variation was influenced by age (*p* = 0.004), but not by tenure (*p* = 0.67).

At the pre-employment check-up, 125 people had normal glycaemia, 45 had impaired fasting glucose (IFG), and there were 8 employees already diagnosed with diabetes. For eight people, these data were missing. The number of people with IFG increased to 58, while those with diabetes rose from 8 to 21. Of the 45 workers with an initial IFG, 10 had currently normal values, 25 still had IFG, and 10 developed diabetes. The difference was statistically significant (chi^2^ = 90.53, *p* < 0.001).

There were several new work-related disorders/symptoms after employment: 56 cases of high blood pressure, 8 patients with heartburns, and 74 drivers with at least one new osteo-articular complaint (24 reported cervical pain, 47 with thoracic pain, and 43 with lumbar pain). Six of them had three localizations of pain, twenty had two localizations and fifty-six had only one concerning segment of the spine.

4. Influence of seniority on the health status of the drivers

The following analysis refers to changes (new onset of symptoms, disorders, and metabolic risk factors) during the monitoring period, from pre-employment to the follow-up check-up.

A statistically significant difference was noted in number of drivers with hypercholesterolemia, which increased from 77 (44% of total) at pre-employment to 113 (64.57%) at the follow-up check-up (chi^2^ = 30.27, *p* < 0.001). The regression models showed no statistically significant influence of tenure on the new diagnosed hypercholesterolemia (Table 2).

At the pre-employment check-up, 125 people had normal glycaemia, 45 had impaired fasting glucose (IFG) and there were 8 employees already diagnosed with diabetes. For eight people, these data were missing. The number of people with IFG increased to 58, while the number of people with diabetes rose from 8 to 21. Of the 45 workers with an initial IFG, 10 had currently normal values, 25 still had IFG and 10 developed diabetes. The difference was statistically significant (chi^2^ = 90.53, *p* < 0.001). Considering only the new onset hyperglycemia, significant correlations were found only between BMI and age (Table 3).

The regression analysis showed that seniority increased the chance of a new onset of high blood pressure by 12% (Table 4), with BMI also having a significant influence in this aspect (Table 4). In order to identify if the metabolically healthy obese drivers are at risk, we calculated the multivariate regression with the same variables in the obesity subgroup. Hypercholesterolemia, blood level of fasting glucose, was not associated with hypertension in this subgroup. The only significant association was tenure (OR = 1.12, 95% CI 1.02–1.23 and *p* = 0.01).

No significant associations were found between any of the variables included in the analysis and pyrosis (Table 5).

The new onset of neck pain was correlated with the female sex and type of vehicle. A higher proportion (32%) of women reported neck pain compared to men (10%). The lower risk in men was maintained even in the multivariate analysis (Table 6).

The highest numbers of neck pain reported were in tram drivers (13 participants, representing 21.31% of the tram drivers), followed by the trolley drivers (7 participants, representing 11.29% of the trolley drivers), and bus (4 participants, representing 6.34% of the bus drivers). The univariate analysis showed an OR = 1.97, but in the multivariate analysis, the association became statistically insignificant.

Interestingly, age seems to be a protective factor for thoracic pain and is independent of other factors (Table 7).

In what concerns the low back pain, it was influenced by seniority and is independent of the other factors considered in this study (Table 8).

## 4. Discussion

The main finding of this retrospective cohort study is that, in public transportation drivers, high blood pressure and low back pain are significantly correlated with tenure, as a rough estimate of the cumulative exposure. The analysis also revealed that these associations were independent of age, gender, and type of vehicle. The results are representative of urban areas’ medium intensity of environmental noise. Also, workers exposure to whole body vibrations was below the European exposure limit.

To the best of our knowledge, this is the first study to specifically look into the correlation between tenure and hypertension in public transportation drivers, despite a well-documented high prevalence of hypertension in this occupation. A meta-analysis estimated a prevalence of high blood pressure in professional drivers in Europe of up to 51% (95% CI: 32–70%) [23]. Other studies found that over 5 years of commercial taxi driving, workers with rather similar exposure, represents a risk factor for hypertension [24]. There are numerous biological explanations for this association. For example, in an experimental study on healthy individuals, the duration of maintenance of the sitting position significantly increases the diastolic blood pressure (DBP), the mean arterial pressure (MAP), the heart rate (HR), and the low-frequency/high-frequency (LF/HF) ratio derived from heart rate variability, reflecting the higher sympathetic nerve activity and peripheral vascular resistance [25]. Reactive oxidative species, which have a pro-atherogenic role [26] are not sufficiently balanced in whole body exposure to vibration [27] or to vehicle brake-derived micro particles [28]. Unbalanced oxidative stress is confirmed in epidemiological studies conducted in different samples of transportation workers [29,30].

Traffic noise might also contribute to high blood pressure. The average noise level identified in Timisoara city should not be in the high risk zone, as the dose response significantly increases only for Lden values above 65 dB [31,32], but we did not have individual noise exposure measurements to exclude this possible influence.

In our cohort, other general risk factors for cardiovascular disease, such as fasting plasma glucose, total cholesterol, and BMI, increased compared to pre-employment check-up, but only BMI maintained an association with hypertension in the multivariate analysis. Interestingly, in the obese subgroup, hypertension was independent of age, sex, hypercholesterolemia, and high fasting glucose, but still directly correlated with tenure. This would imply that even metabolically healthy obese drivers have a high cardiovascular risk, as observed in general population studies [33]. Second, this emphasizes the need for better identification and management of the individual risk in occupational medicine practice from the pre-employment examination, whenever possible. Obesity is influencing heart rate variability (HRV) even in people considered metabolically healthy [34,35]. In this respect, an interesting cohort study found that short-term heart rate variability is predictive of the development of cardiovascular disease in occupational drivers, independent of the traditional risk factors, including BMI [36], and should be considered in the future for better assessing risk in this working population.

Low back pain was also related to tenure in our cohort. For this particular health effect, there are other studies which have also looked at the cumulative effect reflected by tenure, but the results are still controversial. For example, in a meta-analysis, the prevalence of low back pain was 61% (95% CI 0.47–0.74) for drivers of large vehicles, and the risk after more than 5 years of driving was moderate: OR = 2.12 (1.66, 2.69) [37]. In another meta-analysis, the level of evidence was considered low, as 11 studies (among which only two were high quality) showed an association and 12 studies (three were high quality) did not [38]. A strong relation was not found in any of the high-quality studies.

Our study detected a higher risk, which remained the only associated risk in the multivariate analysis. In the above-mentioned meta-analysis [38], causal evidence was found for one of the parameters we had measured, namely the whole-body vibration. The prevalence of 31.7% found in our study is consistent with the estimated prevalence in the literature for the level daily vibration exposure of 0.252 m/s^2^ [39]. In professional drivers, exposure to vibration alongside prolonged sitting and awkward positions are considered to contribute to low back pain. This contrasts with the positive effect of rehabilitation methods using controlled whole body vibration in muscle training [40].

We are aware that stress, an important risk factor for the musculoskeletal symptoms in the general population [41], also applies in terms of occupational stress. However, an interesting meta-analysis which compared physical risk with psychosocial risk regarding the development of low back pain found a higher pooled-OR (1.51, 95% CI 1.14–1.99) for physical risks versus 1.14 (95% CI 1.00, 1.30) for psychosocial risks [42]. In a study targeting drivers, a systematic review found similar results to ours, mainly that the number of years of working as driver is correlated with low back pain in a dose–response relation [43]. This systematic review mentioned that there are few studies in which occupational stress was assessed in relation to low back pain in drivers. One of them mentioned a significant relation with both external (traffic congestion, hostile passenger interaction) and organizational ones: limited time for job breaks and lack of accessibility to the bus [44]. Other recent studies found low back pain to be related to depression, anxiety, and stress [45,46], with the personal, occupational, and client-related burnout [45] or work engagement and sickness presentism [46]. This relation should be further assessed in order to draw a conclusion about its contribution to health issues and the interaction with the complex exposure of public transportation drivers.

We did not find an association between hypercholesterolemia and fasting glucose on one side, and low back pain on the other. In a large cohort study on a general population, with a follow up of 9 years, metabolic factors did not contribute to the development of the low back pain [47]. In other studies, metabolic syndrome was associated with low back pain, mainly in women or in subjects with severe forms of disease [14,48]. Therefore, the lack of association found in our study might be the result of a predominant male sex cohort and the mild to moderate pain, which does not interfere with the working activity.

In the univariate analysis, neck pain was correlated with the type of vehicle (namely was significantly higher in tram drivers) and women. The influence of the type of vehicle was no longer significant when all variables were considered. This was not unexpected, as women were also significantly better represented in the group of tram drivers. The high prevalence in women is consistent with findings in the general population, worldwide, in which point prevalence was higher in women in all age groups [49]. Sedentary behavior [50], awkward positions [51], and stress are the most probable contributors.

There is also a possible connection between the outcomes of drivers’ occupational exposure. Large epidemiological studies gave controversial results about the relation between the cardio-metabolic risk factors and back pain [52,53,54,55]. Besides the common risk factors (e.g., obesity, stress), a bidirectional relation could be considered: while cardiovascular disease might decrease the nutritional supply of the vertebral disks, chronic back pain increases the sympathetic nerves activity and aggravates or initiates a sedentary behavior [55,56]. In order to provide evidence for the causal effect of high blood pressure and type II diabetes on low back pain, a mendelian randomized study was performed [57]. The results of this study showed that a causal effect of systolic and diastolic high blood pressure on back pain and a reverse effect (low back pain as cause of cardiometabolic disease) on type II diabetes. Notably, in this analysis, the outcome was defined by the authors as “back pain associated with health care use”, which implies a certain severity or length of the symptoms.

The results of our study also have some clinical implications, among which the most important is the initiation of the preventive activities from the beginning of employment or as soon as possible. The preventive measures should target both occupational and non-occupational risk factors. Maintaining the hierarchy of the occupational risk control should include engineering (e.g., ergonomic organization of the cabin, reduction in vibration exposure), administrative (proper scheduling of the breaks and of the shifts with enough time to recover), educational (sleep hygiene, stress resilience, diet, smoking cessation, increase in physical activity), and medical monitoring of these possible health issues, and easy access to diagnosis and care in early stages should be provided. In particular, for the medical professionals, identification of subclinical modifications such as endothelial dysfunction [58] or surface electromyography [59] should be tested to conclude if they can add clinical value in this occupational category for an early detection and improved prognosis.

### Limitations

This study has some limitations, one of which is common in longitudinal occupational studies (“the healthy workers effect”). By comparing the initial, pre-employment health status with the current one, we might have missed those who have left the company for exactly the health issues we have been analyzing. We are not able to estimate this effect from our data, but if this would have been the case, and considering that none of the exposure factors have acute effects, these workers will just strengthen the relation between the cumulative exposure and the health effects identified in this study.

In terms of the cardiovascular risk, we did not measure the lipid profile, which would have provided a better prediction of atherosclerosis and hypertension [60] and other diseases related to the metabolic syndrome [61,62,63]. In view of our results, and in accordance with the total workers’ health approach [64], a more comprehensive program should be implemented in drivers for efficient prevention. A multidisciplinary approach, in which a better control of hazards, ergonomic changes, company policy improvement, including promoting productive aging, and better health risk management (e.g., better screening for cardiovascular diseases and targeted interventions) by the occupational medicine doctor, should complement each other [65,66].

Another limitation is that we also did not obtain personal monitoring of noise and vibrations for each participant. However, these working conditions were similar among all of the participants and should not create major differences among them. The good quality of the vibration measurement is reflected in the appropriate estimation of the prevalence of the low back pain.

We are aware that chronic stress might be a possible confounder, as issues such as cardiovascular disease [67], diabetes [68], and musculoskeletal complaints [69] are related to it. In this study, chronic psychosocial stress was assessed; therefore, it might interfere with the significance of tenure of risk factors.

## 5. Conclusions

Professional drivers are exposed to multiple hazards. Tenure is related to the development of cardiovascular and musculoskeletal diseases, suggesting that cumulative exposure contributes to these health issues, beyond the expected effects of aging alone. Therefore, specific interventions, either engineering-related, administrative, educational, or medical should be developed in early stage of the drivers’ career. Occupational-medicine specialists should analyze the contribution of work and non-work related factors for the integrative management of the patient.

## Figures and Tables

**Table 1:** Comparison between pre-employment and follow-up check-up.

Characteristic	Pre-Employment	Follow Up Check Up	*p*
BMI (kg/m^2^) (Average ± SD)	27.69 ± 4.68	30.06 ± 5.2	<0.0001
Total Cholesterol (mg/dL) (Average ± SD)	201.7 ± 39.87	212.62 ± 42.51	0.004
Fasting glucose (mg/dL) (Average ± SD)	96.53 ± 14.94	105.99 ± 20.22	<0.0001
High blood pressure (No, % of total workers)	25 (13.44%)	56 (30.11%)	<0.0001
Neck pain (N, % of total workers)	3 (1.61%)	26 (13.98%)	0.007
Dorsal spine pain (N,% of total workers)	2 (1.08%)	49 (26.34%)	0.02
Lumbar pain (N,% of total workers)	18 (9.67%)	59 (31.72%)	<0.0001

SD = standard deviation.

**Table 2:** Factors influencing the new diagnosed hypercholesterolemia.

	Model 1 *			Model 2 **		
	**OR**	**95% CI**	***p* Value**	**OR**	**95% CI**	***p* Value**
Female sex	1.25	0.47, 3.37	0.65	1.04	0.96, 1.36	0.94
Age (years)	1.02	0.98, 1.06	0.38	1.01	0.99, 1.10	0.66
Seniority (per year)	1.04	0.99, 1.09	0.10	1.04	0.96, 1.11	0.08
BMI	1.03	0.96, 1.10	0.37	1.03	0.98 1.02	0.34
Glycaemia	1.003	0.99, 1.02	0.71	0.99	0.99, 1.02	0.74
Type of vehicle (bus as reference)	0.80	0.53, 1.23	0.31	0.78	0.49, 1.24	0.30

* univariate; ** adjusted to sex, age, seniority, BMI, type of vehicle. Data of BMI, cholesterol, and glycaemia are those recorded at the follow-up check-up.

**Table 3:** Factors influencing the new diagnosed hyperglycemia.

	Model 1 *			Model 2 **		
	**OR**	**95% CI**	***p* Value**	**OR**	**95% CI**	***p* Value**
Female sex	1.26	0.49, 3.21	0.63	1.32	0.47, 3.74	0.59
Age (years)	1.05	1.01, 1.10	0.02	1.05	1.003, 1.09	0.04
Seniority (per year)	1.003	0.96, 1.05	0.91	0.99	0.95, 1.05	0.84
BMI	1.09	1.02, 1.16	0.01	1.08	1.01 1.15	0.02
Hypercholesterolemia	2.09	1.03, 4.23	0.04	1.74	0.83, 3.65	0.14
Type of vehicle (bus as reference)	0.94	0.64, 1.39	0.79	0.99	0.65, 1.52	0.98

* univariate; ** adjusted to sex, age, seniority, BMI, type of vehicle. Data of BMI, cholesterol, and glycaemia are those recorded at the follow-up check-up.

**Table 4:** Factors influencing newly diagnosed high blood pressure.

	Model 1 *			Model 2 **		
	OR	95% CI	*p* Value	OR	95% CI	*p* Value
Female sex	0.6	0.25, 1.43	0.25	0.47	0.16, 1.36	0.16
Age (years)	1.06	1.01, 1.09	0.007	1.04	0.99, 1.1	0.15
Seniority (per year)	1.11	1.06, 1.16	<0.0001	1.12	1.06, 1.18	0.00003
BMI	1.14	1.06, 1.22	0.00006	1.17	1.08, 1.26	0.00007
Cholesterol	1.0005	0.99, 1.01	0.15	1.001	0.4, 1.96	0.79
Glycaemia	1.01	1.0003, 1.03	0.045	1.003	0.99, 1.02	0.72
Type of vehicle (bus as reference)	0.98	0.67, 1.45	0.93	0.86	0.54, 1.38	0.53

* univariate; ** adjusted to sex, age, seniority, BMI, type of vehicle. Data of BMI, cholesterol, and glycaemia are those recorded at the follow-up check-up.

**Table 5:** Factors correlated to new diagnosed pyrosis.

	Model 1 *			Model 2 **		
	**OR**	**95% CI**	***p* Value**	**OR**	**95% CI**	***p* Value**
Female sex	0.6	0.12, 3.01	0.55	0.71	0.13, 3.93	0.69
Age (years)	1.0003	0.93, 1.08	0.99	0.99	0.91, 1.08	0.96
Seniority (per year)	1.03	0.94, 1.1	0.46	1.01	0.94, 1.12	0.56
Actual BMI	0.95	0.83, 1.07	0.37	0.95	0.83, 1.07	0.44
Type of vehicle (bus as reference)	1.4	0.63, 3.12	0.4	1.26	0.53, 2.98	0.6

* univariate; ** adjusted to sex, age, seniority, BMI, type of vehicle.

**Table 6:** Factors correlated to the new onset neck pain.

	Model 1 *			Model 2 **			Model 3 ***		
	OR	95% CI	95% CI	*p*	95% CI	*p*	OR	95% CI	*p*
Sex (female as reference)	0.25	0.09, 0.67	0.008	0.30	0.11, 0.89	0.03	0.3	0.1, 0.89	0.03
Age (years)	1.03	0.97, 1.08	0.33	1.02	0.96, 1.08	0.49	1.02	0.95, 1.09	0.57
Seniority per (year)	1.04	0.99, 1.1	0.14	1.03	0.98, 1.1	0.23	1.03	0.97, 1.1	0.34
Actual BMI	0.99	0.92, 1.08	0.98	1.007	0.93, 1.09	0.87	0.99	0.91, 1.1	0.94
Type of vehicle (bus as reference)	1.97	1.12, 3.44	0.01	1.63	0.88, 3.01	0.12	1.64	0.87, 3.07	0.13
High blood pressure							2.3	0.79, 6.7	0.13
Total Cholesterol							0.99	0.99, 1.008	0.64
Fasting glycaemia							0.99	0.96, 1.01	0.32

* univariate; ** adjusted to sex, age, seniority, BMI, type of vehicle; *** adjusted to high blood pressure, total cholesterol, and fasting glycaemia recorded at the follow-up check-up.

**Table 7:** Factors correlated to the new onset thoracic pain.

	Model 1 *			Model 2 **			Model 3 ***		
	OR	95% CI	*p*	OR	95% CI	*p*	OR	95% CI	*p*
Sex (female as reference)	1.41	0.5, 3.99	0.51	1.41	0.47, 4.25	0.54	1.4	0.45, 4.26	0.56
Age (years)	0.94	0.9, 0.98	0.003	0.95	0.91, 0.99	0.02	0.93	0.89, 0.98	0.007
Seniority per (year)	0.95	0.91, 1.007	0.07	0.97	0.92, 1.03	0.36	0.97	0.92, 1.02	0.28
Actual BMI	0.98	0.92, 1.04	0.53	0.99	0.92, 1.05	0.68	0.96	0.9, 1.04	0.33
Type of vehicle (bus as reference)	0.94	0.63, 1.41	0.76	0.96	0.62, 1.5	0.87	0.97	0.61, 1.52	0.88
High blood pressure							2.22	0.95, 5.17	0.07
Total Cholesterol							0.99	0.99, 1.007	0.8
Fasting glycaemia							0.99	0.98, 1.02	0.95

* univariate; ** adjusted to sex, age, seniority, BMI, type of vehicle; *** adjusted to high blood pressure, total cholesterol, and fasting glycaemia recorded at the follow-up check-up.

**Table 8:** Factors correlated to the new onset low back pain.

	Model 1 *			Model 2 **			Model 3 ***		
	OR	95% CI	*p*	OR	95% CI	*p*	OR	95% CI	*p*
Sex (female as reference)	0.79	0.31, 2.05	0.63	0.72	0.26, 2.03	0.54	0.76	0.27, 21.15	0.6
Age (years)	1.02	0.98, 1.06	0.26	1.007	0.96, 1.05	0.75	1.008	0.96, 1.06	0.74
Seniority per (year)	1.07	1.02, 1.12	0.003	1.07	1.02, 1.13	0.005	1.07	1.02, 1.13	0.006
Actual BMI	1.02	0.95, 1.09	0.6	1.02	0.95, 1.09	0.5	1.01	0.94, 1.09	0.66
Type of vehicle (bus as reference)	1.02	0.67, 1.54	0.91	0.91	0.58, 1.44	0.69	0.88	0.56, 1.4	0.6
High blood pressure	1.68	0.85, 3.31	0.13				1.5	0.67, 3.35	0.31
Total cholesterol	1.2	0.59, 2.43	0.62				1.09	0.51, 2.31	0.83
Fasting glycaemia	0.99	0.98, 1.01	0.55				0.99	0.98, 1.006	0.19

* univariate; ** adjusted to sex, age, seniority, BMI, type of vehicle *** adjusted to high blood pressure, total cholesterol, and fasting glycaemia recorded at the follow-up check-up.

## Data Availability

Data available from the main author.
